# Seasonality Effects on the Mineral Profile of Goats Farmed in the Semiarid Region of Brazil

**DOI:** 10.3390/vetsci8010008

**Published:** 2021-01-06

**Authors:** Joseney Maia Lima, Rodolfo Gurgel Vale, Rejane dos Santos Sousa, Talyta Lins Nunes, Jucélio da Silva Gameleira, Jerson Marques Cavalcante, Antonio Humberto Hamad Minervino, Enrico Lippi Ortolani, Raimundo Alves Barrêto Júnior

**Affiliations:** 1 Department of Animal Science, Federal Rural University of the Semiarid Region, UFERSA. Av. Francisco Mota, s/n—Bairro Presidente Costa e Silva, Mossoró CEP 59625-900, RN, Brazil; joseneylima@hotmail.com (J.M.L.); rodolfo_g_vale@hotmail.com (R.G.V.); talyta_lins@hotmail.com (T.L.N.); jucelio_gameleira@hotmail.com (J.d.S.G.); jersonmarques@hotmail.com (J.M.C.); 2 Department of Clinical Science, College of Veterinary Medicine and Animal Science, FMVZ, University of Sao Paulo, USP. Av. Orlando Marques de Paiva, 87, Cidade Universitária, São Paulo CEP 05508-270, SP, Brazil; rejane.santossousa@gmail.com (R.d.S.S.); ortolani@usp.br (E.L.O.); 3 Laboratory of Animal Health, LARSANA, Federal University of Western Pará, UFOPA. Rua Vera Paz, s/n, Salé, Santarém CEP 68040-255, PA, Brazil

**Keywords:** deficiency, minerals, caprine, seasonal

## Abstract

Seasonality effects on the mineral profile of goats were evaluated. Fifty males were divided into two groups, one with mineral supplementation and one control. Seasonal evaluation was conducted during four periods: beginning, middle, and end of the dry period and middle of the rainy period. Rib and liver biopsies were performed, and blood was collected at each period to evaluate mineral accumulation. Ca, P, Cu, Fe, Mo, Zn, and Co concentrations were determined using inductively coupled plasma optical emission spectrometry after acid digestion. Normal Ca, P, and Mo; low Cu, Zn, and Co; and high Fe levels were observed in the diet. The young animals analyzed showed normal serum and bone Ca and P concentrations, suggesting no need for supplementation throughout the entire year under the conditions of this study. Iron showed high values throughout the year, being potentially dangerous especially owing to its antagonistic relationship with other elements. Cu and Zn deficiency in the diet was observed under the conditions of this study, requiring supplementation with values higher than those contained in the mineral supplement used in the middle and end of the dry period. The supply of specific mineral supplement formulated for animals farmed in the semiarid region is suggested and would reduce costs.

## 1. Introduction

Goat farming is a global activity, with Brazil having approximately 9.3 million heads of which more than 90% is concentrated in the northeast region [1]. In this region, the Caatinga biome predominates in 60% of the area, with semiarid climate (low humidity and rainfall) and xerophilous vegetation consisting of small shrub and tree species, commonly deciduous and with thorns [2]. Forage production in the *Caatinga* is strongly influenced by seasonality, with higher production in the rainy season and deficiency in the drought period, which markedly changes the chemical composition and quality of these plants [3].

Minerals are inorganic substances, vital to ruminants and present in all tissues and fluids, having essential functions such as being part of tissue structure and supporting acid-base balance, osmotic pressure, and cell membrane permeability [4]. In an extensive breeding system, mineral deficiency is one of the most harmful limitations, mainly as it affects the productive and reproductive performance of the animals [5]. This deficiency can vary from mild to severe and presents more or less characteristic clinical signs, which can be detected through analysis of the mineral profile of the animals to show which minerals are deficient and when to supplement them [6]. The analysis of mineral levels in body fluids and tissues like the serum, liver, and bone assists in the accurate deduction of the occurrence of mineral deficiencies using a small sample size [7].

Goats are animals adapted to consume a wide variety of plants, presenting a feeding behavior that can be classified as opportunistic and easily modifying their feeding preferences according to forage availability and season [8]. These animals also graze over long distances, which leads some researchers to believe that they can supply themselves nutritionally when there is availability of native pasture without grazing area limitation. Knowledge regarding mineral deficiencies in goats is quite limited. To our knowledge, there are no studies evaluating the interaction between seasonality and mineral supplementation in goats grazing in the *Caatinga* biome and thus the importance of conducting this study. Thus, we aimed to evaluate the blood and tissue mineral profile of young goats raised in the Caatinga system through the different seasons and submitted or not to mineral supplementation.

## 2. Materials and Methods

This study included 50 castrated male goats of undefined breed, aged 6 to 8 months, dewormed, and kept on an extensive farm in the city of Mossoró, RN, Brazil. This research was approved by the ethics committee on the use of animals of the Federal Rural University of the Semi-Arid (Process No. 23091.004703/2013-63).

The design used was entirely random, distributed in a 2 × 4 factorial arrangement (two treatments and four periods of the year). The goats were previously selected and distributed in two groups with 25 animals each, a treatment group—with mineral supplementation at will (previously adapted with Caprinofós^®^), and a control group—without mineral supplementation. Goats had access to large areas of Caatinga during the day but return to a collective elevated pen where they stayed for the nighttime. The animals in the treatment group had access to mineral mixture offered at feeders placed inside the elevated pen. Goats were offered the mineral mixture from the end of the day until dawn of the next day (approximately 12–14 h). During this time, the mineral mixture was offered ad libitum with sufficient feeders for all animals. 

**Figure 1:**
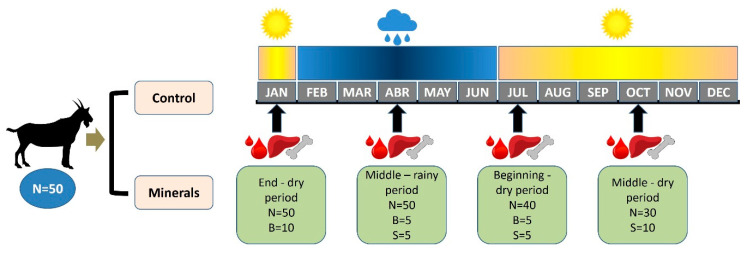
Study design. Colors bars indicate the seasons (yellow: dry season; blue: rainy season). N (number) defines the total number of goats in the experiment at that timepoint. B (biopsy): indicate the number of animals sampled by biopsy (liver or rib) at the period. S (slaughtered): indicate the number of animals slaughtered at each period for tissue sampling. Blood, liver, and bone icons represent the tissues sampled at each period. Control: goats without mineral supplementation; Minerals: treated group that received mineral supplementation.

The seasonal evaluation was conducted during one year in four different months according to the seasons: January, April, July, and October. The region has two well defined seasons, the dry season (July to January) and the rainy season (February to June). We sampled at defined periods: end of the dry period (January), middle of the rainy period (April), beginning of the dry period (July), and middle of the dry period (October). The Figure 1 presents the experimental design. 

Blood, liver, and rib samples were collected from 10 animals in each group at each seasonal period to evaluate the mineral profile. The tissue samples were collected from five slaughtered animals and from five animals submitted to surgical intervention (biopsies). The blood was collected by jugular venipuncture using vacuum tubes without anticoagulant, which were centrifuged at 1032× *g* for 5 min to obtain serum, which was stored in plastic microtubes at −20 °C for subsequent analysis. Liver biopsy was performed as described by Minervino et al. [9] and bone biopsy (rib) according to the classic methodology [10]. The samples were stored in sterile cryovials and frozen at −20 °C for subsequent analysis.

Fragments of 1 g of tissue (liver and bone) were dried in an oven at 103 °C for 24 h. Next, fragments weighing 0.2 to 0.4 g were subjected to the acid digestion process (nitric-perchloric) in a digestion block and subsequently analyzed for mineral concentrations using inductively coupled plasma optical emission spectrometry (ICP-OES, Varian^®^, São Paulo, Brazil) at the Laboratory of Nutritional and Metabolic Diseases of the School of Veterinary Medicine and Animal Husbandry (USP) [11].

The percentages of Ca and P were determined in bone tissue based on dry matter. Cu, Fe, Zn, and Mo concentrations were determined in the liver and Ca, P, Cu, Fe, Zn, Co, and Mo concentrations were quantified in the serum.

The statistical analysis was performed using SAS 9.3 software (SAS institute Inc., Cary, NC, USA). The experimental data were analyzed following the principles of homogeneity of variances and normal distribution using the Shapiro–Wilk test and subsequently subjected to analysis of variance using the PROC GLM procedure to evaluate the differences between experimental groups. The Tukey’s range test was used to compare seasonal periods within the same group using the PROC ANOVA procedure. Differences were considered significant when *p* ≤ 0.05. 

## 3. Results and Discussion

Table 1 shows the means and standard deviations of mineral element concentrations in serum. Table 2 shows the means and standard deviations of mineral element concentrations in bone and liver tissues.

Serum Ca levels remained within the normal range for goats (Reference: 8.2 to 11.7 mg/dL) in both the treated and control groups and in all seasonal periods, consistent with the findings in other studies [12,13], with lower serum concentrations in the control group than those in the treatment group only at the end of the dry period. The animals in the control and treatment groups showed serum Ca values above the reference values at the beginning of the dry period, maybe owing to the greater mobilization of this element from bone tissue, which can be confirmed by lower bone calcium levels in this period. Bone Ca concentration was similar to levels generally found in goats [14,15] but below bone concentrations obtained elsewhere [16].

Serum P concentrations in both groups and in all seasonal periods remained above the reference values (4.2 to 9.1 mg/dL) [13], except in the middle of the rainy season. High serum P levels are related to the age of the animals; the animals in the current study were young. Young goats have higher serum P concentrations than adult animals [17] because of a greater efficiency in the intestinal absorption and renal reabsorption of Ca to maintain bone development in these animals [18]. The percentage of bone P levels in this study are consistent with levels reported in some other studies [14,15] and are below the levels reported by Lengarite et al. (2012). According to Suttle [6], Ca and P concentrations are more accurate when evaluated in bone tissue. In young animals, the ideal Ca:P ratio should vary from 1:1 to 2:1, with a tendency to gradually increase until adulthood [17,19]. In this study, the bone Ca:P ratio varied between 2:1 and 2.1:1 in all periods, indicating that both the control and supplemented animals had enough of this element for growth.

Similar Ca and P (*p* > 0.05) bone percentages were observed in the control and treated groups in all seasonal periods; a difference between these elements was observed only in the serum but with satisfactory values compared to reference levels for the caprine species, which suggests that there is no need for Ca and P supplementation for goats in this age group, farmed in the studied region, and grazing on native pasture.

In this study, except for the beginning of the dry period, the control group showed excess serum Fe in all periods studied. The treated group, in contrast, had a serum concentration above the normal range only in the middle of the dry period. The normal serum concentration of Fe in goats ranges from 17 to 36 µmol/L, and a concentration above 39 µmol/L is considered excess [6]. Liver Fe concentrations were above the normal range for sheep; however, they were consistent with those reported in other studies on goats in northern Kenya [16] and in northeastern Brazil, with values around 150 ppm in Pernambuco [20], Ceará [21], and Rio Grande do Norte [22]. The highest serum Fe values were seen in the middle of the dry period, and the highest liver concentrations were seen at the end of the dry period, a consequence of the intake in the previous period. The dryness of the land associated with more wind and forage scarcity in these periods may have resulted in increased involuntary soil intake, and consequently, increased serum and liver Fe concentrations. Involuntary soil intake can vary from 10 to 25% of dry matter in the diet of sheep grazing in arid lands [6].

Higher serum Cu concentrations were found in the treatment group than those in the control group in the middle and end of the dry period, associated with high values in the other two periods, suggest the need for Cu supplementation. Serum levels from 3 to 9 μmol/L are considered the limit for Cu deficiency in goats [6]. Thus, as shown in this study, at the end of the dry period, the animals in the control group presented serum levels within the critical range and the treatment group presented higher levels. In other studies, these values varied between 10.54 and 19.98 μmol/L [20,23,24,25].

Liver Cu levels in both groups and during all seasonal periods were below the levels reported by several other studies, wherein values of between 150 and 328 ppm were observed [16,20,26]. However, there was an important difference between the treated and control animals; the Cu levels in control animals were 34% below those of the treatment animals in the same periods. It should also be noted that considering the normal range for the species, the control group presented extremely low levels which were very close to those mentioned by Radostits et al. [27] for secondary Cu deficiency (15 to 19 ppm). Cu levels above 101 ppm are considered normal, and levels between 0 and 50 ppm indicate deficiency, which would place the control animals at least in the phase of liver depletion for the element [6]. Excess of Fe in the diet can cause Cu deficiency in man and animals [28] and was already described in sheep in the same region [22]. High dietary Fe interferes with Cu availability for uptake by animal tissues [28].

In addition to the possibility of low Cu concentrations in the *Caatinga* soil and plants, several minerals interfere with Cu absorption, such as a high dietary amount of Mo, S, Fe, Cd, Se, and Zn [29]. In the present study, Zn and Mo levels, important Cu antagonists, were below or within the normal range; in contrast, excess serum and liver Fe levels were detected. Moreover, goats are known for the habit of feeding preferentially on legumes, which are rich in proteins that can reduce Cu absorption [6]. All these factors may have contributed to the observed Cu deficiency.

Mo is an essential mineral for all animal species; however, in ruminants its importance is almost exclusively associated with secondary Cu deficiency, but the reference minimum serum and hepatic values of this element for goats were not found in any previous studies. Serum Mo concentrations varied between 0.11 ± 0.04 and 0.71 ± 0.41 µmol/L in this study. Studies on goats, and mainly on sheep, have reported serum levels between 0.28 ± 0.11 and 0.8 ± 0.19 µmol/L [20,26,30] according to the content of this element in the diet. Serum levels reported by Marques et al. [21] in goats in the northeast (0.28 ± 0.11 μmol/L) were much higher than those found in the two seasonal periods in the control group (0.11 ± 0.04 μmol/L at the end of dry season and 0.15 ± 0.09 μmol/L in the middle of the rainy season). However, they were consistent with findings from a study by Pott et al. [31] who reported similar concentrations, suggesting that these levels may be close to those found in animals in the stage of depletion or deficiency. Liver Mo concentration remained within the normal range (2 to 4 ppm) as reported by Suttle [6] but below the levels found in Pernambuco (6.5 ppm) by Marques et al. [21]. In general, the levels were similar to the data reported for sheep in other studies [26,31]. The observed difference (*p* ≤ 0.05) in both serum and liver Mo concentrations between the treated and control groups showed the positive effect of Mo supplementation.

Serum Zn levels were at levels defined as deficient, except in the middle of the dry period, when the serum Zn concentration was within the normal range. Serum Zn concentrations between 12.2 to 18.2 μmol/L are considered normal for goats [27]. Liver concentration analysis is the safest method to diagnose Zn deficiency [32]. In this study, liver Zn values were below the normal range (101 to 200 ppm) in three of the eight evaluations in the groups and seasonal periods [6], sometimes including animals from the treated group. This indicated the need for Zn supplementation greater than the amount contained in the mineral supplement, mainly in the beginning and middle of the dry period. Zn interacts with several minerals in both absorption and excretion [11] and the Zn bioavailability in ruminants appear to be affected by dietary factors that have not been yet clearly defined [33]. Excessive dietary Fe can interfere with Zn absorption in humans and other species and this Zn status may be related with the excess of iron observed in goat liver in the present study [28]. 

The control group showed serum Co levels below the range considered normal (0.17 to 0.51 µmol/L; [28]) at the end of the dry period and in the middle of the rainy period. Serum Co concentrations in the treated group were within the normal range in all evaluated periods. These results show Co deficiency in the diet of goats grazing on native pastures in the semiarid region and the need for Co supplementation in the diet. Studies on Co deficiency in goat serum are scarce; however, these results show the possibility of using this evaluation to diagnose the deficiency of this element.

## 4. Conclusions

Young goat showed normal serum and bone Ca and P concentrations, suggesting no need for supplementation in all the seasons throughout the year in the Caatinga environment. Iron showed high values throughout the year, being potentially dangerous especially owing to its antagonistic relationship with other elements, especially Cu.

Cu and Zn deficiency in the diet was observed under the conditions of this study, requiring supplementation, especially in the middle and end of the dry period, with higher amounts of Cu and Zn than those included in the mineral supplement used. The mineral supplementation used was efficient to maintain ideal serum Co levels and was required at the end of the dry and in the middle of the rainy periods.

A specific mineral supplement with seasonal variation in composition can be used for animals farmed under the conditions of this study, thereby reducing supplementation costs.

## Figures and Tables

**Table 1:** Mean values and standard deviations of serum concentrations of calcium, phosphorus, copper, iron, molybdenum, zinc, and cobalt in goats reared on native pasture supplemented (Treatment) or not (Control) with mineral mixture during different seasonal periods in the semiarid region.

Variable	Groups	Season			
		End of Dry Period	Middle of Wet Period	Beginning of Dry Period	Middle of Dry Period
Ca (mg/dL)	Control	9.3 ± 1.1 ^B,c,^*	10.8 ± 0.8 ^b^	12.4 ± 0.3 ^a^	11.3 ± 0.9 ^b^
	Treatment	11.3 ± 1.3 ^A,a,b^	10.7 ± 1.2 ^b^	12.5 ± 0.5 ^a^	11.3 ± 0.9 ^a,b^
P (mg/dL)	Control	10.9 ± 2.5 ^a,b^	8.7 ± 1.4 ^b^	9.9 ± 1.8 ^a,b^	12.7 ± 2.2 ^a^
	Treatment	9.4 ± 2.2 ^b,c^	8.9 ± 3.0 ^c^	11.1 ± 2.3 ^a,b^	12.7 ± 0.7 ^a^
Cu (µmol/L)	Control	8.2 ± 1.4 ^B^	9.9 ± 1.6	9.7 ± 0.97	10.1 ± 1.4 ^B^
	Treatment	10.7 ± 1.1 ^A^	11.1 ± 2.4	9.8 ± 1.4	12.2 ± 1.7 ^A^
Fe (µmol/L)	Control	39.2 ± 9.1 ^a,b^	42.2 ± 10.6 ^A,a,b^	33.9 ± 13.0 ^b^	52.6 ± 5.1 ^a^
	Treatment	30.0 ± 9.2 ^b^	33.5 ± 5.9 ^Bab^	36.7 ± 10.1 ^a,b^	44.0 ± 6.1 ^a^
Mo (µmol/L)	Control	0.1 ± 0.0 ^B,b^	0.15 ± 0.1 ^b^	0.30 ± 0.1 ^B,b^	0.55 ± 0.2 ^a^
	Treatment	0.4 ± 0.1 ^A,a,b^	0.27 ± 0.12 ^b^	0.47 ± 0.1 ^A,a,b^	0.71 ± 0.4 ^a^
Zn (µmol/L)	Control	10.5 ± 1.5 ^b^	11.7 ± 1.3 ^b^	11.6 ± 2.3 ^b^	14.8 ± 2.1 ^a^
	Treatment	9.6 ± 2.9 ^b^	10.9 ± 1.8 ^b^	10.9 ± 1.3 ^b^	14.9 ± 1.8 ^a^
Co (µmol/L)	Control	0.2 ± 0.1 ^B,b^	0.11 ± 0.04 ^b^	0.35 ± 0.14 ^a,b^	0.5 ± 0.2 ^a^
	Treatment	0.4 ± 0.1 ^A,b^	0.22 ± 0.14 ^b^	0.36 ± 0.1 ^b^	0.68 ± 0.28 ^a^

* Uppercase letters in the column indicate difference between groups and lowercase letters in the row indicate difference between seasonal periods.

**Table 2:** Mean values and standard deviations of bone concentrations of calcium and phosphorus and liver concentrations of copper, iron, molybdenum, and zinc in goats reared on native pasture supplemented (Treatment) or not (Control) with mineral mixture during different seasonal periods in the semiarid region.

Variables	Groups	Season			
		End of Dry Period	Middle of Wet Period	Beginning of Dry Period	Middle of Dry Period
Ca (bone) (%)	Control	20.90 ± 1.3 ^a,^*	21.16 ± 2.1 ^a^	18.64 ± 2.9 ^b^	22.09 ± 1.5 ^a^
	Treatment	21.94 ± 2.2 ^a^	22.72 ± 0.9 ^a^	17.15 ± 2.5 ^b^	21.80 ± 3.8 ^a^
P (bone) (%)	Control	9.72 ± 0.5 ^a^	10.10 ± 1.3 ^a^	8.86 ± 1.5 ^b^	10.53 ± 0.7 ^a^
	Treatment	10.14 ± 0.9 ^a^	10.66 ± 1.4 ^a^	7.95 ± 1.2 ^b^	10.14 ± 1.9 ^a^
Cu (liver) (ppm)	Control	21.63 ± 21.1 ^B^	33.43 ± 14.5 ^B^	22.98 ± 12.6 ^B^	21.21 ± 26.6 ^B^
	Treatment	75.27 ± 36.2 ^A^	122.96 ± 44.4 ^A^	68.83 ± 43.9 ^A^	94.18 ± 76.8 ^A^
Fe (liver) (ppm)	Control	191.0 ± 78.8 ^a^	128.9 ± 19.8 ^b^	121.78 ± 34.3 ^b^	123.39 ± 28.8 ^b^
	Treatment	173.4 ± 70.1 ^a^	146.5 ± 25.6 ^a,b^	118.37 ± 40.1 ^b^	126.44 ± 37.7 ^b^
Mo (liver) (ppm)	Control	3.23 ± 1.2 ^a^	2.32 ± 0.7 ^B,b^	2.92 ± 1.0 ^B,a,b^	2.73 ± 0.6 ^B,a,b^
	Treatment	4.83 ± 0.9	4.99 ± 0.7 ^A^	4.71 ± 0.7 ^A^	4.66 ± 0.97 ^A^
Zn (liver) (ppm)	Control	109.93 ± 16.8 ^a^	107.09 ± 14.9 ^a^	104.26 ± 15.9 ^a^	85.76 ± 13.6 ^b^
	Treatment	104.74 ± 14.8	103.03 ± 13.4	97.59 ± 17.3	92.96 ± 9.9

* Uppercase letters in the column indicate differences between groups and lowercase letters indicate differences between seasonal periods.

## Data Availability

The raw data from this research is available upon request to the corresponding author.
